# Epidemiology and Incidence of Surgically Treated ACL Injuries in Division I Collegiate Athletes

**DOI:** 10.1177/23259671261418054

**Published:** 2026-03-27

**Authors:** Thomas J. Kremen, Andromahi Trivellas, Varun Sriram, Grant G. Schroeder, David R. McAllister

**Affiliations:** † Department of Orthopaedic Surgery, David Geffen School of Medicine, University of California, Los Angeles, Los Angeles, California, USA; ‡ Synergy Orthopaedics, San Diego, California, USA; § Renaissance School of Medicine, Stony Brook University, Stony Brook, New York, USA; ‖ Stanford University School of Medicine, Stanford, California, USA; Investigation performed at the David Geffen School of Medicine, University of California, Los Angeles, Los Angeles, CA

**Keywords:** anterior cruciate ligament, Division I collegiate athletes, incidence rate ratio, sex differences, sport-related injury

## Abstract

**Background:**

Anterior cruciate ligament (ACL) injuries are common in collegiate athletes. This study aimed to quantify the incidence of ACL injuries among athletes across all sports in the Pacific-12 Conference (Pac-12) from 2016 to 2021.

**Purpose/Hypothesis:**

To evaluate the epidemiology of ACL injuries in a major Division I collegiate athletic conference and assess the influence of sex, sport, playing surface, and time of season on ACL injury. It was hypothesized that athletes with clinically significant ACL injuries would be treated surgically.

**Study Design:**

Case series; Level of evidence, 4.

**Methods:**

Data were analyzed from the Pac-12 Health Analytics Program sports injury registry. All athletes competing at the Division I level in the Pac-12 conference who had sustained an isolated ACL tear and undergone reconstruction between 2016 and 2021 were included in this study. Athletes with non-ACL-medial collateral ligament multiligamentous injuries, those who had a reported ACL injury but no surgery, and those with incomplete data were excluded. Variables included sex (sex assigned at birth), sport, playing surface, event occurrence, year of injury, season of injury, and mechanism of injury, and were analyzed. Incidence rates (IR) were calculated based on injuries recorded compared with the number of athletes per sport per year. The incidence rate ratio (IRR) was then computed comparing female to male incidence rates in sports for which this was applicable.

**Results:**

A total of 313 ACL injuries in 286 athletes (148 women, 165 men) met the inclusion criteria for this study. The overall mean IR of ACL injuries per athlete-year was 1.1%. The highest IR was found in female soccer players (5%). Among male athletes, the highest IR was in football at 1.9%. Statistically significant differences in IRR were identified in gymnastics (3.1), softball/baseball (6), and soccer (2.9). Most ACL injuries occurred in-season via a noncontact mechanism.

**Conclusion:**

In this cohort of athletes, most ACL injuries occurred in-season via a noncontact mechanism, and the overall mean IR of ACL injuries per athlete-year was 1.1%. Among male athletes, football had the highest incidence of ACL injury. Female athletes were at significantly higher risk of ACL injury than their male counterparts in gymnastics, softball, and soccer, with soccer having the highest incidence.

Anterior cruciate ligament (ACL) injuries can be career-altering or career-ending for athletes of all skill levels. While much has been published on ACL injuries and surgeries in general, there remains a scarcity of literature on factors contributing to ACL injuries among elite National Collegiate Athletic Association (NCAA) Division 1 collegiate athletes. Among this group, ACL injuries can be career-transforming injuries due to their associated disability. In fact, ACL injuries have been noted to be one of the most common injuries in collegiate athletics.^20^

Many studies have reported on ACL injuries in athletes across different sports at the high school level.^12,21,25^ However, data at the elite level are significantly limited, with only a few studies reporting on injuries among European soccer players^13^ or American National Football League (NFL) players.^20^ There have been a handful of reports describing ACL injuries observed among NCAA athletes participating in specific sports or a select number of sports,^1,4,8,17^ and at least 1 report of a cross-section of many sports,^2^ or a systematic review encompassing many sports.^18^ However, the existing literature on this topic, specific to the elite NCAA athlete population, is relatively limited. This is particularly the case when characterizing ACL injuries across all sports and institutions within a highly competitive NCAA Division 1 conference. Nevertheless, ACL reconstruction (ACLR) remains one of the most common orthopaedic sports medicine procedures, with the incidence rising each year.^10^ This work reports on the incidence of ACL injuries in female and male athletes across all sports in the Pacific-12 Conference (Pac-12) conference from the years 2016 to 2021 and highlights differences in in-season injuries, playing surface, and sport.

It has previously been reported that young female athletes are at a 3-fold higher risk of tearing their ACLs than their male counterparts.^22^ However, it remains unknown whether this was specific to certain sports and whether this was true across all sports at the NCAA Division 1 level, where athletes likely have more rigorous demands about training and competitions. Furthermore, high-risk sports have previously been identified at the high school level—including women's soccer, basketball, gymnastics, and lacrosse,^12,25^ and it is unknown whether these risks persist at the elite collegiate level.

The Pac-12 Health Analytics Program sports injury registry (or Pac-12 Sports Injury Research Archive) is a database initiated in 2016 to prospectively collect injury data from all athletes in the Pac-12 conference. It is a comprehensive deidentified database from which select variables specific to individual research inquiries may be requested. Additional schools and sports were added to the original registry after 2016.

This study aimed to define the incidence of ACL injuries among NCAA Division I athletes stratified by sport, sex (sex assigned at birth), mechanism of injury, in-season versus off-season, competition versus practice, and playing surface. We hypothesized that observed ACL injury events would differ significantly across sports, playing surfaces, competition seasons, and sexes.

## Methods

### Data Collection

The study population comprised NCAA Division 1 athletes in the Pac-12 Conference across all sports and the 12-member universities during the observation period, which varied depending on when each school began recording data in the Pac-12 Sports Injury Research Archive. The observation period was from July 2016 through 11 November 2021 for 9 Pac-12 schools; from July 2018 through 11 November 2021 for 1 school; from July 2019 through 11 November 2021 for 1 school; and from July 2021 through 11 November 2021 for 1 school. During the period of observation, common data elements were entered into the Pac-12 Sports Injury Research Archive for all health events—defined as a player presenting to their athletic trainer or physician with a health complaint—suffered by an athlete. Most of the data were entered into the database by athletic trainers, with initial data entry occurring nearly in real time, daily. Advanced imaging was not recorded in the database. The recorded data did include a deidentified athlete identifier, athlete sex, athlete sport, event diagnosis, whether the event required surgical intervention, event season, event mechanism, event setting, whether it was a first-time event (primary injury) versus a recurrence, event playing surface, and event year, as well as other information not relevant to the present study. The event mechanism and associated details were witnessed by and recorded on the day of injury by the team athletic trainer, certified (ATC). This included documentation of in-season vs. off-season based on the NCAA-defined in-season time frame for each sport. Even ACL injuries treated by physicians outside the institution attended by each athlete were captured in the database, as their longitudinal rehabilitation and participation in team activities would have been performed to a large degree at the institution in which the athlete was enrolled.

After the determination of institutional review board exemption for this study and approval of our Pac-12 Health Analytics Program application, all ACL injuries in the Pac-12 Sports Injury Research Archive during the observation period were queried among all athletes who consented to having their deidentified injury and illness data used for research.

Separate from the Pac-12 Sports Injury Research Archive data extraction, the number of athletes appearing on publicly available team rosters for all Pac-12 sports and schools was recorded for all relevant observation period years. Certain sports—including men's rugby, women's field hockey, equestrian, and synchronized swimming—are sports only designated as varsity sports at individual schools. To deidentify participants, the data collected for these sports were aggregated and presented here as *unique sports*.

### Data Processing and Statistical Analysis

Athletes with multiligamentous knee injuries (MLKIs) were excluded from the analysis unless that multiligamentous injury was a concurrent ACL-medial collateral ligament (MCL) injury, as these injuries are typically managed in a manner equivalent to isolated ACL injuries. In addition, ACL injuries for which no surgery was reported were excluded from the analysis, as the vast majority of athletes participating in NCAA Division 1 athletics would elect to undergo surgical treatment for a complete or a partial ACL tear with significant instability symptoms. ACL injuries treated nonsurgically were excluded from analysis due to these injuries being presumed to be either partial ACL injuries that did not significantly affect knee biomechanics or were incorrectly labeled as ACL injuries. Similarly, given that patients with ACL injuries have numerous interactions with athletic trainers over the course of their recovery as well as interactions with physical therapists, physicians and other allied health personnel capable of capturing data, ACL injuries with incomplete data sets were excluded from the analysis due to concern for these injuries not representing clinically significant ACL injuries or the injuries being incorrectly labeled as ACL injuries. Given the infrequent nature of MLKIs and the stratification of our extracted data by sex, sport, and year, to maintain participant deidentification, detailed data on non-ACL-MCL MLKI patients and patients with ACL injuries who did not undergo operative treatment were kept in aggregate and not acquired for analysis.

Statistical analysis was performed using Excel Version 16.49 (Microsoft) in consultation with the biostatistics core facility at our institution. *Events* were defined as the number of ACL tears during a particular period. *Athletes affected* is defined as the number of athletes suffering from at least one ACL tear during a particular period. The *Incidence rate* (*IR*) was calculated by dividing the number of events by the number of relevant *athlete-years*. Since the Pac-12 Sports Injury Research Archive database does not include individual-athlete exposure data, such as the number of hours in practice or competition, an athlete-year in this study is defined as an athlete appearing on a team roster for a given year. The *incidence rate ratio* (*IRR*), calculated by dividing the female incidence rate by the male incidence rate, was used to compare IRs between female and male athletes, with statistically significant differences defined as IRR 95% CIs not including 1 (*P* < .05).

## Results

The observation period included 29,036 athlete-years of exposure involving 12,449 male and 16,587 female athletes participating in 18 different sports—including 4 unique sports. A total of 445 ACL injuries were recorded during the observation period, which represented a gross IR of 1.5% (95% CI, 0.0139-0.0167). Of these 445 ACL injuries, 132 were characterized as either a non-ACL-MCL MLKI, as not requiring surgical intervention, or were associated with incomplete data sets, and thus were excluded from further analysis. Incomplete data sets were noted in 11 patients. After application of these exclusion criteria, 313 surgically treated ACL tears were identified during the observation period (Table 1). Among female athletes, 148 ACL tears occurred in 137 athletes, for an overall IR of 1.2% per athlete-year. Among male athletes, 165 ACL tears occurred in 149 athletes, for an overall IR of 1.1% per athlete-year. For female athletes, soccer had the highest IR of 5% per athlete-year, followed by basketball (2.7%) and gymnastics (2.4%). For male athletes, football has the highest IR of 1.9% per athlete-year, followed by soccer (1.7%) and basketball (1.4%). Female athletes had a significantly higher rate of ACL tears than male athletes in soccer (IRR, 2.9 [95% CI, 1.6-5.2]) and gymnastics (IRR, 3.1 [95% CI, 1.0-9.3]). Although volleyball was observed to have an IRR of 2.6 (95% CI, 0.33-20.5), this was not statistically significant, likely due to the relatively low number of injuries, particularly among men (n = 1), compared with women (n = 9). There was no statistically significant difference in ACL tear rates between female and male athletes across all sports combined (IRR, 1.2 [95% CI, 0.96-1.5]).

**Table 1:** ACL Tears by Sport*^*a*^*

	Overall				Female Athletes				Male Athletes				
	Events, n	Athletes Affected, n	Athlete Exposures, n	Incidence Rate (95% CI)*^*b*^*	Events, n	Athletes Affected, n	Athlete Exposures, n	Incidence Rate (95% CI)*^*b*^*	Events, n	Athletes Affected, n	Athlete Exposures, n	Incidence Rate (95% CI)*^*b*^*	IRR (95% CI)*^*c*^*
Football	118	108	6113	0.0193 (0.0158 to 0.0228)	–	–	–	–	118	108	6113	0.0193 (0.0158 to 0.0228)	–
Soccer	89	77	2268	0.0392 (0.0311 to 0.0474)	76	68	1518	0.0501 (0.0388 to 0.0613)	13	9	750	0.0173 (0.0079 to 0.0268)	2.8885 (1.604 to 5.2018)*^*d*^*
Basketball	31	29	1570	0.0197 (0.0128 to 0.0267)	19	17	716	0.0265 (0.0146 to 0.0385)	12	12	854	0.014 (0.0061 to 0.022)	1.8886 (0.9168 to 3.8906)
Gymnastics	19	18	1125	0.0169 (0.0093 to 0.0245)	15	14	616	0.0243 (0.012 to 0.0367)	4	4	509	0.0079 (0.0002 to 0.0156)	3.0989 (1.0285 to 9.3372)*^*d*^*
Lacrosse	10	10	972	0.0103 (0.0039 to 0.0167)	10	10	972	0.0103 (0.003 to 0.0167)	–	–	–	–	–
Volleyball	10	10	1140	0.0088 (0.0033 to 0.0142)	9	9	885	0.0102 (0.0035 to 0.0168)	1	1	255	0.0039 (−0.0038 to 0.0116)	2.5921 (0.3284 to 20.4605)
Softball	9	9	870	0.0103 (0.0036 to 0.0171)	9	9	870	0.0103 (0.0036 to .0171)	–	–	–	–	–
Unique sports	9	8	3815	0.0024 (0.0008 to 0.0039)	4	4	1908	0.0021 (0 to 0.0042)	5	4	1908	0.0026 (0.0003 to 0.0049)	0.8 (0.2148 to 2.9792)
Track and field	6	6	4359	0.0014 (0.0003 to 0.0025)	2	2	2426	0.0008 (−0.0003 to 0.002)	4	4	1933	0.0021 (0 to	0.3984 (0.073 to 2.175)
Baseball	3	3	1745	0.0017 (−0.0002 to 0.0037)	–	–	–	–	3	3	1745	0.0017 (−0.0002 to 0.0037)	–
Wrestling	3	2	511	0.0059 (−0.0008 to 0.0125)	–	–	–	–	3	2	511	0.0059 (−0.0008 to 0.0125)	–
Skiing	2	2	241	0.0083 (−0.0032 to 0.0198)	2	2	117	0.0171 (−0.0066 to 0.0409)	0	0	124	0 (0 to 0)	–
Swimming and diving	2	2	1672	0.0012 (−0.0005 to 0.0029)	0	0	941	0 (0 to 0)	2	2	731	0.0027 (−0.0011 to 0.0065)	–
Cross country	1	1	1756	0.0006 (−0.0005 to 0.0017)	1	1	1025	0.001 (−0.0009 to 0.0029)	0	0	731	0 (0 to 0)	–
Tennis	1	1	879	0.0011 (−0.0011 to 0.0034)	1	1	455	0.0022 (−0.0021 to 0.0065)	0	0	424	0 (0 to 0)	–
Total	313	286	29036	0.0108 (0.0096 to 0.012)	148	137	12449	0.0119 (0.01 to 0.0138)	165	149	16587	0.0099 (0.0084 to 0.0115)	1.1951 (0.9573 to 1.492)

a Unique sports included men's rugby, women's field hockey, equestrian, and synchronized swimming, which are sports only designated as varsity sports at individual schools. These are aggregated to deidentify participants. IR, incidence rate; IRR, incidence rate ratio.

b IR per 1 athlete-year.

c IRR, female versus male athletes.

d Statistical significance (*P* < 05).

Considering all sports combined (Table 2), a total of 222 ACL tears occurred in-season, 71 occurred in the off-season, and 20 did not have seasonality specified. A total of 229 injuries occurred via a noncontact mechanism, 69 via a contact mechanism, and 15 did not have a mechanism specified. There were 254 primary ACL injuries (also known as a first injury), 58 injuries were recurrent, and 1 was not specified. The IR ranged from 0.9% to 1.6% per athlete-year for years 2016-2017 to 2021-2122. Compared with male athletes, female athletes had significantly higher rates of ACL tear in competition (IRR, 1.7 [95% CI, 1.2-2.5]), on a constructed surface (IRR, 4 [95% CI, 1.6-10.1]), and on an organic surface (IRR, 1.9 [95% CI, 3-2.7]).

**Table 2:** All sports–ACL Tears by Season, Mechanism, Setting, Recurrence, Surface, and Year*^*a*^*

		Overall			Female Athletes			Male Athletes			
		Events, n	Athletes Affected, n	Incidence Rate (95% CI)*^*b*^*	Events, n	Athletes Affected, n	Incidence Rate (95% CI)*^*b*^*	Events, n	Athletes Affected, n	Incidence Rate (95% CI)*^*b*^*	IRR (95% CI)*^*c*^*
Season	In-season	222	208	0.0076 (0.0066 to 0.0087)	106	100	0.0085 (0.0069 to 0.0101)	116	108	0.007 (0.0057 to 0.0083)	1.2175 (0.9356 to 1.5844)
	Not specified	20	20	0.0007 (0.0004 to 0.001)	13	13	0.001 (0.0005 to 0.0016)	7	7	0.0004 (0.0001 to 0.0007)	2.4744 (0.9872 to 6.202)
	Off-season	71	69	0.0024 (0.0019 to 0.003)	29	28	0.0023 (0.0015 to 0.0032)	42	41	0.0025 (0.0018 to 0.0033)	0.92 (0.5731 to 1.4767)
Mechanism	Contact	69	65	0.0024 (0.0018 to 0.0029)	33	31	0.0027 (0.0017 to 0.0036)	36	34	0.0022 (0.0015 to 0.0029)	1.2213 (0.7615 to 1.9588)
	Non-contact	229	215	0.0079 (0.0069 to 0.0089)	108	100	0.0087 (0.007 to 0.0103)	121	115	0.0073 (0.006 to 0.0086)	1.1892 (0.9175 to 1.5415)
	Not specified	15	15	0.0005 (0.0003 to 0.0008)	7	7	0.0006 (0.0001 to 0.001)	8	8	0.0005 (0.0001 to 0.0008)	1.1658 (0.4228 to 3.215)
Setting	Competition	108	104	0.0037 (0.003 to 0.0044)	61	59	0.0049 (0.0037 to 0.0061)	47	45	0.0028 (0.002 to 0.0036)	1.7293 (1.1821 to 2.5297)*^*d*^*
	Not specified	40	40	0.0014 (0.001 to 0.0018)	20	20	0.0016 (0.0009 to 0.0023)	20	20	0.0012 (0.0007 to 0.0017)	1.3324 (0.7169 to
	Practice	165	155	0.0057 (0.0048 to 0.0065)	67	63	0.0054 (0.0041 to 0.0067)	98	92	0.0059 (0.0047 to 0.0071)	0.9109 (0.6676 to 1.2428)
Recurrence	First injury	254	245	0.0087 (0.0077 to 0.0098)	119	118	0.0096 (0.0078 to 0.0113)	135	127	0.0081 (0.0068 to 0.0095)	1.1745 (0.9179 to 1.5027)
	Not specified	1	1	0 (0 to 0.0001)	0	0	0 (0 to 0)	1	1	0.0001 (0 to 0.0002)	–
	Recurrence	58	52	0.002 (0.0015 to	29	27	0.0023 (0.0015 to 0.0032)	29	25	0.0017 (0.0011 to 0.0024)	1.3324 (0.7963 to 2.2293)
Surface	Constructed surface	24	21	0.0008 (0.0005 to 0.0012)	18	16	0.0014 (0.0008 to 0.0021)	6	5	0.0004 (0.0001 to 0.0007)	3.9971 (1.5867 to 10.0697)*^*d*^*
	Not specified	30	30	0.001 (0.0007 to 0.0014)	12	12	0.001 (0.0004 to 0.0015)	18	18	0.0011 (0.0006 to 0.0016)	0.8883 (0.4279 to 1.844)
	Organic surface	110	101	0.0038 (0.0031 to 0.0045)	64	59	0.0051 (0.0039 to 0.0064)	46	42	0.0028 (0.002 to 0.0036)	1.8537 (1.2691 to 2.7076)*^*d*^*
	Synthetic	149	143	0.0051 (0.0043 to 0.006)	54	52	0.0043 (0.0032 to 0.0055)	95	91	0.0057 (0.0046 to 0.0069)	0.7574 (0.5423 to 1.0577)
Year	2016-17	43	43	0.009 (0.0063 to 0.0117)	24	24	0.012 (0.0072 to 0.0169)	19	19	0.0068 (0.0038 to 0.0099)	1.7621 (0.9652 to 3.2168)
	2017-18	50	49	0.0104 (0.0075 to 0.0133)	25	25	0.0119 (0.0072 to 0.0165)	25	24	0.0092 (0.0056 to 0.0129)	1.2877 (0.7397 to 2.2416)
	2018-19	55	55	0.0104 (0.0076 to 0.0131)	30	30	0.0132 (0.0085 to 0.0179)	25	25	0.0083 (0.005 to 0.0115)	1.5912 (0.9359 to 2.7054)
	2019-20	62	62	0.0107 (0.0081 to 0.0134)	26	26	0.0104 (0.0064 to 0.0144)	36	36	0.011 (0.0074 to 0.0145)	0.9521 (0.5749 to 1.5767)
	2020-21	65	65	0.0109 (0.0083 to 0.0136)	29	29	0.0114 (0.0072 to 0.0155)	36	36	0.0106 (0.0071 to 0.014)	1.0766 (0.6602 to 1.7557)
	2021-22	38	38	0.0157 (0.0107 to 0.0207)	14	14	0.0135 (0.0064 to 0.0206)	24	24	0.0173 (0.0104 to 0.0242)	0.7821 (0.4046 to 1.5118)
Total		313	286	0.0108 (0.0096 to 0.012)	148	137	0.0119 (0.01 to 0.0138)	165	149	0.0099 (0.0084 to 0.0115)	1.1951 (0.9573 to 1.492)

a ACL, anterior cruciate ligament; IR, incidence rate; IRR, incidence rate ratio.

b IR per 1 athlete-year.

c IRR, female versus male athletes.

d Statistical significance (*P* < .05).

Among soccer athletes (Table 3), there were 89 ACL tears, 76 in female and 13 in male athletes, with an overall IR of 3.9% (95% CI, 0.0311-0.0474). Female athletes had a significantly higher overall rate of ACL tear compared with male athletes (IRR, 2.9 [95% CI, 1.6-5.2]), particularly for in season ACL injuries (IRR, 4.7 [95% CI, 1.9-11.9]), injuries via a noncontact mechanism (IRR, 2 [95% CI, 1-3.9]), injuries occurred in competition (IRR, 18.3 [95% CI, 2.5-133.2]), as a first injury, that is primary ACL tear (IRR, 7.8 [95% CI, 2.8-21.4]), and on an organic surface (IRR, 4.2 [95% CI, 1.8-9.8]).

**Table 3:** Soccer–ACL Tears by Season, Mechanism, Setting, Recurrence, and Surface*^*a*^*

		Overall			Female Athletes			Male Athletes			
		Events, n	Athletes Affected, n	Incidence rate (95% CI)*^*b*^*	Events, n	Athletes Affected, n	Incidence rate (95% CI)*^*b*^*	Events, n	Athletes Affected, n	Incidence Rate (95% CI)*^*b*^*	IRR (95% CI)*^*c*^*
Season	In-season	53	50	0.0234 (0.0171 to 0.0297)	48	45	0.0316 (0.0227 to 0.0406)	5	5	0.0067 (0.0008 to 0.0125)	4.7432 (1.8883 to 11.9147)*^*d*^*
	Not specified	12	12	0.0053 (0.0023 to 0.0083)	11	11	0.0072 (0.003 to 0.0115)	1	1	0.0013 (0 to 0.0039)	5.4349 (0.7017 to 42.098)
	Off-season	24	22	0.0106 (0.0063 to 0.0148)	17	16	0.0112 (0.0059 to 0.0165)	7	6	0.0093 (0.0024 to 0.0162)	1.1999 (0.4976 to 2.8935)
Mechanism	Contact	26	25	0.0115 (0.0071 to 0.0159)	26	25	0.0171 (0.0105 to	0	0	0 (0 to 0)	–
	Non-contact	56	48	0.0247 (0.0182 to 0.0312)	45	40	0.0296 (0.021,0.0383)	11	8	0.0147 (0.006,0.0233)	2.0213 (1.0455,3.9078)*^*c*^*
	Not specified	7	7	0.0031 (0.0008 to 0.0054)	5	5	0.0033 (0.0004 to 0.0062)	2	2	0.0027 (0 to 0.0064)	1.2352 (0.2396 to 6.3668)
Setting	Competition	38	36	0.0168 (0.0114 to 0.0221)	37	35	0.0244 (0.0165 to 0.0322)	1	1	0.0013 (0 to 0.0039)	18.2811 (2.5082 to 133.2435)*^*d*^*
	Not specified	12	12	0.0053 (0.0023 to 0.0083)	9	9	0.0059 (0.0021 to 0.0098)	3	3	0.004 (0 to 0.0085)	1.4823 (0.4013 to 5.4752)
	Practice	39	34	0.0172 (0.0118 to 0.0226)	30	28	0.0198 (0.0127 to 0.0268)	9	6	0.012 (0.0042 to 0.0198)	1.6469 (0.7819 to 3.4689)
Recurrence	First injury	67	66	0.0295 (0.0225 to 0.0366)	63	62	0.0415 (0.0313 to 0.0518)	4	4	0.0053 (0.0001 to 0.0106)	7.7818 (2.8325 to 21.3793)*^*d*^*
	Not specified	0	0	0 (0 to 0)	0	0	0 (0 to 0)	0	0	0 (0 to 0)	–
	Recurrence	22	19	0.0097 (0.0056 to 0.0138)	13	13	0.0086 (0.0039 to 0.0132)	9	6	0.012 (0.0042 to 0.0198)	0.7137 (0.3051 to 1.6696)
Surface	Constructed surface	0	0	0 (0 to 0)	0	0	0 (0 to 0)	0	0	0 (0 to 0)	–
	Not specified	6	6	0.0026 (0.0005 to 0.0048)	5	5	0.0033 (0.0004 to 0.0062)	1	1	0.0013 (0 to 0.0039)	2.4704 (0.2886 to 21.1462)
	Organic surface	57	50	0.0251 (0.0186 to 0.0317)	51	46	0.0336 (0.0244 to 0.0428)	6	4	0.008 (0.0016 to 0.0144)	4.1997 (1.8023,9.7859)*^*d*^*
	Synthetic	26	25	0.0115 (0.0071 to 0.0159)	20	20	0.0132 (0.0074 to 0.019)	6	5	0.008 (0.0016 to 0.0144)	1.6469 (0.6614 to 4.1011)
Total		89	78	0.0392 (0.0311 to 0.0474)	76	69	0.0501 (0.0388 to 0.0613)	13	9	0.0173 (0.0079 to 0.0268)	2.8885 (1.604 to 5.2018)*^*d*^*

a ACL, anterior cruciate ligament; IR, incidence rate; IRR, incidence rate ratio.

b IR per 1 athlete-year.

c IRR, female versus male athletes.

d Statistical significance (*P* < .05).

For football (Table 4), a total of 118 ACL tears were observed, yielding an incidence rate of 1.9% per athlete-year. Of these, 87 ACL tears occurred in-season, 26 occurred in the off-season, and 5 did not have seasonality specified. Regarding setting, 72 injuries occurred in practice, 36 in competition, and 10 were not specified. A total of 102 injuries were a first injury, 15 were a recurrence, and 1 was not specified.

**Table 4:** Football–ACL Tears by Season, Mechanism, Setting, Recurrence, and Surface*^*a*^*

		Overall		
		Events, n	Athletes Affected, n	Incidence Rate (95% CI)*^*b*^*
Season	In-season	87	81	0.0142 (0.0112 to 0.0172)
	Not specified	5	5	0.0008 (0.0001 to 0.0015)
	Off-season	26	26	0.0043 (0.0026 to 0.0059)
Mechanism	Contact	31	29	0.0051 (0.0033 to 0.0069)
	Noncontact	81	78	0.0133 (0.0104 to 0.0161)
	Not specified	6	6	0.001 (0.0002 to 0.0018)
Setting	Competition	36	34	0.0059 (0.004 to 0.0078)
	Not specified	10	10	0.0016 (0.0006 to 0.0026)
	Practice	72	70	0.0118 (0.0091 to 0.0145)
Recurrence	First injury	102	94	0.0167 (0.0134 to 0.0199)
	Not specified	1	1	0.0002 (0 to 0.0005)
	Recurrence	15	14	0.0025 (0.0012 to 0.0037)
Surface	Constructed surface	0	0	0 (0 to 0)
	Not specified	11	11	0.0018 (0.0007 to 0.0029)
	Organic surface	36	34	0.0059 (0.004 to 0.0078)
	Synthetic	71	69	0.0116 (0.0089 to 0.0143)
Total		118	108	0.0193 (0.0158 to 0.0228)

a ACL, anterior cruciate ligament; IR, incidence rate.

b IR per 1 athlete-year.

Among basketball athletes (Table 5), there were 31 ACL tears, with 27 of those occurring in-season (16 injuries in practice, 11 in competition), 3 occurred in the off-season, and 1 did not have seasonality specified. Overall, there was not a significant difference in the rate of ACL tear for female versus male athletes (IRR, 1.9 [95% CI, 0.9-3.9]).

**Table 5:** Basketball–ACL Tears by Season, Mechanism, Setting, Recurrence, and Surface*^*a*^*

		Overall			Female			Male			
		Events, n	Athletes Affected, n	Incidence Rate (95% CI)*^*b*^*	Events, n	Athletes Affected, n	Incidence Rate (95% CI)*^*b*^*	Events, n	Athletes Affected, n	Incidence Rate (95% CI)*^*b*^*	IRR (95% CI)*^*c*^*
Season	In-season	27	26	0.0172 (0.0107 to 0.0237)	16	15	0.0223 (0.0114 to 0.0333)	11	11	0.0129 (0.0053 to 0.0205)	1.735 (0.8052 to 3.7385)
	Not specified	1	1	0.0006 (0 to 0.0019)	1	1	0.0014 (0 to 0.0041)	0	0	0 (0 to 0)	–
	Off-season	3	3	0.0019 (0 to 0.0041)	2	2	0.0028 (0 to 0.0067)	1	1	0.0012 (0 to 0.0035)	2.3856 (0.2163 to 26.3099)
Mechanism	Contact	8	7	0.0051 (0.0016 to 0.0086)	6	5	0.0084 (0.0017 to 0.0151)	2	2	0.0023 (0 to 0.0056)	3.5784 (0.7222 to 7.7298)
	Non-contact	23	22	0.0146 (0.0087 to 0.0206)	13	12	0.0182 (0.0083 to 0.028)	10	10	0.0117 (0.0045 to	1.5506 (0.6799 to 3.5363)
	Not specified	0	0	0 (0 to 0)	0	0	0 (0 to 0)	0	0	0 (0 to 0)	–
Setting	Competition	11	11	0.007 (0.0029 to 0.0111)	7	7	0.0098 (0.0025 to 0.017)	4	4	0.0047 (0.0001 to 0.0093)	2.0874 (0.611 to 7.1307)
	Not specified	4	4	0.0025 (0.0001 to 0.005)	3	3	0.0042 (0 to 0.0089)	1	1	0.0012 (0 to 0.0035)	3.5784 (0.3722 to
	Practice	16	16	0.0102 (0.0052 to 0.0152)	9	9	0.0126 (0.0044 to	7	7	0.0082 (0.0021 to 0.0143)	1.5336 (0.5711 to 4.118)
Recurrence	First injury	22	22	0.014 (0.0082 to 0.0199)	12	12	0.0168 (0.0073 to 0.0262)	10	10	0.0117 (0.0045 to 0.019)	1.4314 (0.6184 to 3.313)
	Not specified	0	0	0 (0 to 0)	0	0	0 (0 to 0)	0	0	0 (0 to 0)	–
	Recurrence	9	7	0.0057 (0.002 to 0.0095)	7	5	0.0098 (0.0025 to 0.017)	2	2	0.0023 (0 to 0.0056)	4.1748 (0.8672 to 20.0968)
Surface	Constructed surface	0	0	0 (0 to 0)	0	0	0 (0 to 0)	0	0	0 (0 to 0)	–
	Not specified	1	1	0.0006 (0 to 0.0019)	1	1	0.0014 (0 to 0.0041)	0	0	0 (0 to 0)	–
	Organic surface	0	0	0 (0 to 0)	0	0	0 (0 to 0)	0	0	0 (0 to 0)	–
	Synthetic	30	28	0.0191 (0.0123 to 0.0259)	18	16	0.0251 (0.0135 to 0.0367)	12	12	0.014 (0.0061 to 0.022)	1.7892 (0.8618 to 3.7144)
Total		31	29	0.0197 (0.0128 to 0.0267)	19	17	0.0265 (0.0146 to 0.0385)	12	12	0.014 (0.0061 to 0.022)	1.8886 (0.9168 to 3.8906)

a ACL, anterior cruciate ligament; IR, incidence rate; IRR, incidence rate ratio.

b IR per 1 athlete-year.

c IRR, female versus male patients.

For softball and baseball combined (Table 6), a total of 12 ACL injuries were observed, with 11 occurring in-season and 1 with seasonality unspecified. All 12 injuries were noncontact and first-time injuries. The IR ranged from 0.9% to 1.6% per athlete-year for years 2016-2017 to 2021-2022. Female softball players had a significantly higher rate of ACL tears compared with male baseball players in-season (IRR, 9 [95% CI, 1.9-41.7]) and during practice (IRR, 12 [95% CI, 1.4-99.9]). Overall, female softball players had a significantly higher rate of ACL tears than male baseball players (IRR, 6 [95% CI, 1.6-22.2]).

**Table 6:** Softball and Baseball–ACL Tears by Season, Mechanism, Setting, Recurrence, and Surface*^*a*^*

		Overall			Softball			Baseball			
		Events, n	Athletes Affected, n	Incidence Rate (95% CI)*^*b*^*	Events, n	Athletes Affected, n	Incidence Rate (95% CI)*^*b*^*	Events, n	Athletes Affected, n	Incidence Rate (95% CI)*^*b*^*	IRR (95% CI)*^*c*^*
Season	In-season	11	11	0.0042 (0.0017 to 0.0067)	9	9	0.0103 (0.0036 to 0.0171)	2	2	0.0011 (0 to 0.0027)	9.0199 (1.9488 to 41.7471)*^*d*^*
	Not specified	1	1	0.0004 (0 to 0.0011)	0	0	0 (0 to 0)	1	1	0.0006 (0 to 0.0017)	–
	Off-season	0	0	0 (0 to 0)	0	0	0 (0 to 0)	0	0	0 (0 to 0)	–
Mechanism	Contact	0	0	0 (0 to 0)	0	0	0 (0 to 0)	0	0	0 (0 to 0)	–
	Non-contact	12	12	0.0046 (0.002 to 0.0072)	9	9	0.0103 (0.0036 to 0.0171)	3	3	0.0017 (0 to 0.0037)	6.0132 (1.6279 to 22.2119)*^*d*^*
	Not specified	0	0	0 (0 to 0)	0	0	0 (0 to 0)	0	0	0 (0 to 0)	–
Setting	Competition	5	5	0.0019 (0.0002 to 0.0036)	3	3	0.0034 (0 to 0.0073)	2	2	0.0011 (0 to 0.0027)	3.0066 (0.5024 to 17.9941)
	Not specified	0	0	0 (0 to 0)	0	0	0 (0 to 0)	0	0	0 (0 to 0)	–
	Practice	7	7	0.0027 (0.0007 to 0.0047)	6	6	0.0069 (0.0014 to	1	1	0.0006 (0 to 0.0017)	12.0265 (1.4478 to 99.8982)*^*d*^*
Recurrence	First injury	12	12	0.0046 (0.002 to 0.0072)	9	9	0.0103 (0.0036 to 0.0171)	3	3	0.0017 (0 to 0.0037)	6.0132 (1.6279 to 22.2119)*^*d*^*
	Not specified	0	0	0 (0 to 0)	0	0	0 (0 to 0)	0	0	0 (0 to 0)	–
	Recurrence	0	0	0 (0 to 0)	0	0	0 (0 to 0)	0	0	0 (0 to )	–
Surface	Constructed surface	0	0	0 (0 to 0)	0	0	0 (0 to 0)	0	0	0 (0 to 0)	–
	Not specified	0	0	0 (0 to 0)	0	0	0 (0 to 0)	0	0	0 (0 to 0)	–
	Organic surface	9	9	0.0034 (0.0012 to 0.0057)	7	7	0.008 (0.0021 to 0.014)	2	2	0.0011 (0 to 0.0027)	7.0154 (1.4573 to 33.7712)*^*d*^*
	Synthetic	3	3	0.0011 (0 to 0.0024)	2	2	0.0023 (0 to 0.0055)	1	1	0.0006 (0 to 0.0017)	4.0088 (0.3635 to 44.2121)
Total		12	12	0.0046 (0.002 to 0.0072)	9	9	0.0103 (0.0036 to 0.0171)	3	3	0.0017 (0 to 0.0037)	6.0132 (1.6279 to 22.2119)*^*d*^*

a ACL, anterior cruciate ligament; IR, incidence rate; IRR, incidence rate ratio.

b IR per 1 athlete-year.

c IRR, female versus male athletes.

d Statistical significance (*P* < .05).

## Discussion

The present study reports on surgically treated ACL injuries among intercollegiate athletes across all sports at all participating schools in a major, highly competitive NCAA Division I collegiate athletic conference over 5 years. Therefore, we believe our characterization of ACL injuries among elite intercollegiate athletes will help inform sports medicine professionals about normative values for future comparisons and potentially optimize injury-prevention strategies.

Overall, in our cohort of elite NCAA Division I intercollegiate athletes, we observed that ACL injuries most often occurred in-season via a noncontact mechanism, with an aggregate mean IR of 1.1 ACL injuries per athlete-year. Across all sports, there was no significant difference in the IRR of ACL injuries between female and male athletes (IRR, 1.2). However, there was a significant difference in IRR between female and male athletes participating in soccer (IRR, 2.9) and gymnastics (IRR, 3.1), with both sports showing a higher ACL injury rate among female athletes. In addition, women's softball had a significantly higher IRR of ACL injuries (IRR, 6) compared with the similar sport of men's baseball. The highest IR for ACL injuries was found in female soccer players, with an IR of 5%, whereas the highest IR in male athletes was observed among football players at 1.9%. Numerous previous studies have attempted to illuminate the cause of the discrepancy between sex-based differences in ACL injury risk without much success. Studies have posed the possibilities of differences in conditioning, anatomy, hormone levels, flexibility, proprioception, athletic facilities, and more as being potential contributors to this observed difference, with no definitive conclusion being reached.^19^

In our study, the distribution of injuries throughout the athletic season showed a higher in-season incidence rate of ACL injuries than during the off-season. This difference may be explained by the likely increase in competition and training intensity during the athletic season relative to the off-season. A previous study of fatigue and ACL injuries in NCAA athletes found that the early part of the season had the highest incidence of ACL injuries, further supporting the idea that the transition to higher-intensity competition increases the likelihood of an athlete suffering an ACL injury.^3^

The playing surface has remained an area of interest regarding ACL injuries, especially in sports played on multiple surfaces (eg, football). When stratifying by sport, football athletes observed the same significant increase in ACL injury IR when playing on synthetic surfaces compared with organic surfaces. Previous studies of NCAA football athletes also found an increased risk of ACL injury on synthetic surfaces compared with organic surfaces.^9,15^ We did not find soccer athletes who displayed this finding; instead, we observed a lower injury IR on synthetic surfaces than on organic surfaces. Softball athletes exhibited a pattern similar to soccer, with a lower injury IR on synthetic surfaces than on organic surfaces. These observations, taken together, suggest that the playing surface, while certainly a factor, is not solely responsible for differences in ACL injury risk among collegiate athletes, with sport-specific motions potentially contributing to these differences. Previous studies have not reached a consensus on whether organic or synthetic surfaces pose a higher risk of ACL injuries. Notably, in a review of 10 studies on ACL injury and playing surface, among the 4 studies that observed an increased risk of ACL injury on synthetic surfaces, all were football cohorts, one of which observed a reduced injury risk on synthetic surfaces.^5^ Moreover, 1 study of NFL athletes observed a higher incidence of noncontact ACL injuries on organic surfaces during competition, and a higher incidence of noncontact ACL injuries on synthetic surfaces during practice, further supporting the complexity of this observation.^23^

An overwhelming majority of reported ACL injuries were noted to occur via a noncontact injury mechanism rather than contact injuries. Of the 298 total injuries with specified mechanism, nearly 77% were noncontact injuries. This is consistent with a previous study that found about 75% of ACL injuries were noncontact.^6^ Although not fully understood, noncontact ACL injuries are frequently stated as occurring during deceleration or landing from a jump, actions in which the quadriceps place a significant degree of strain on the ACL.^14^ Across all sports in which >10 ACL injuries per athlete year were observed (football, soccer, basketball, gymnastics, and softball + baseball), soccer and basketball had the highest IR of noncontact injuries. The injury mechanisms believed to be strongly associated with noncontact ACL injuries (deceleration/sharp changes in direction and landing from a jump) are extremely common motions in these 2 sports.

The recurrence rate of ACL injuries among athletes remains an important and concerning outcome. Significantly, beyond the associated time away from competition, athletes suffering recurrent ACL injuries are likely to have more severe degenerative radiographic changes compared with those suffering primary ACL tears.^11^ In a study of ACL injuries in NCAA athletes between 2004 and 2014, male athletes were observed to have a significantly higher rate of recurrent ACL ruptures when compared with female athletes.^11^ Our study did not observe such a trend with the difference in the IRs of recurrent ACL injuries between male (IR, 0.0017) and female (IR, 0.0023) athletes showing no significant difference (IRR, 1.3324 [95% CI, 0.7963-2.2293]). Among soccer players, we observed a significantly higher rate of primary injuries among female athletes than male athletes (IRR, 7.7818 [95% CI, 2.8325-21.3793]). The same trend was observed among baseball and softball athletes (IRR, 6.0132 [95% CI, 1.6279, 22.2119]). No differences in IRs of recurrent ACL injuries were observed across any sport with >10 injuries.

A noteworthy advantage of the present study is that it captures a unique dataset that would likely be very difficult to reproduce in the future. Although NCAA injury data exists, it includes all divisions, which may not apply to elite collegiate athletes, as there are likely differences in skill level, training, pre-collegiate risk exposure, et cetera, across NCAA divisions. Furthermore, since allowing 1-time immediate NCAA eligibility for all student-athletes in the transfer portal in 2021 and expanding it again in 2024, there has been a much higher rate of athlete turnover from one year to the next on any given intercollegiate athletics team. This high turnover of athletes can lead to data capture among the same athlete over time being less consistent than in the years before the transfer portal. Thus, the data included in this study are a highly valuable evaluation of ACL injury risk in the elite NCAA Division 1 collegiate athlete population.

### Limitations

The most notable limitation of this study is the 132 injuries recorded as ACL injuries that were excluded from our analysis. The excluded patients had injuries characterized as non-ACL-MCL MLKI, were not treated with surgical intervention, or had incomplete data sets. Also, >90% of these 132 injuries in this study were characterized as either non-ACL-MCL MLKIs or as being treated nonoperatively. Our inability to quantify the number of patients in these 2 categories (non-ACL-MCL MLKI vs nonoperatively treated) is a noteworthy limitation of this study. However, our decision that the 132 excluded injuries characterized in this manner are composed of non-ACL-MCL MLKI injuries and partial ACL injuries is consistent with what others have reported in the literature for this type of injury. LaPrade RF et al^16^ in a prospective study of 187 acute knee injuries presenting with hemarthrosis, noted that acute ACL tears occurred in 74% (136 of 187) of patients, and, of those ACL tears, 18% (24 of 136) were associated with non-ACL-MCL multiple ligament injuries. If we apply this prospective data to the 445 reported ACL injuries identified in our data, approximately 15% (~67 ACL injuries) of the reported ACL injuries would be non-ACL-MCL MLKIs, which is about half of the 132 excluded injuries. A comprehensive review of the literature characterizing partial ACL tears demonstrates that 10% to 27% of ACL injuries are partial tears.^7^ If we applied this to our data conservatively by estimating that 20% (~89 ACL injuries) of the reported ACL injuries identified in our study were likely partial ACL tears, that would account for more than half of the 132 excluded injuries. Stone et al^24^ reviewed the literature on the management of partial ACL tears in studies with >4-year follow-up. They noted that 9% to 78% of nonoperatively treated partial ACL tears noted persistent instability, and 21% to 66% of patients returned to sports participation. Based on the noted studies, a composite of partial ACL injuries managed nonoperatively, and non-ACL-MCL MLKIs could account for more than the 132 ACL injuries excluded from analysis in this study. Nevertheless, these assumptions remain a noteworthy limitation of our results.

This study also has several other limitations, including the fact that data captured in the Pac-12 sports injury database were entered by individuals from a variety of allied health backgrounds, most commonly ATCs with varying levels of experience. Injuries were recorded daily in near-real time. Typically, data were entered before any physician verification and before any advanced imaging. Thus, there is potential for knee injuries initially misdiagnosed as ACL injuries. For example, if an athlete with a medial collateral ligament sprain complains of acute knee pain and instability from an injury that occurred during an off-campus competition, and the ATC's initial impression is an ACL tear, that injury is recorded as an ACL tear in the database without any advanced imaging being obtained. Potentially, no physical examination will be performed by an experienced clinician/physician. In this case, an MCL sprain will be captured in the database as an ACL tear. Although downstream correction of provisional diagnoses was possible in the Pac-12 sports injury data entry interface, not all diagnoses entered were confirmed by a physician. Despite this limitation, an ACL-deficient athlete is highly unlikely to compete at an elite collegiate level in a cutting and pivoting sport. Whereas if an ACL injury truly did occur, that injury will very likely have been confirmed by advanced imaging (eg, magnetic resonance imaging) and will certainly have been confirmed on examination by an orthopaedic surgeon before proceeding with an ACLR procedure. Thus, our assumption to exclude ACL injuries captured in the database that were not treated surgically is likely appropriate, particularly since the data in this study were collected before the implementation of the transfer portal, that is, transfer out of an institution (and potentially outside of the Pac-12 Sports Injury Research Archive catchment area) was much less common. In light of this, we would expect the vast majority of true complete ACL tears to have been treated surgically and for those procedures to have been captured in the database. To ensure the confidentiality of athlete-protected health information, the Pac-12 Sports Injury Research Archive data were provided for analysis in aggregate, deidentified form; thus, confirmation of true, complete ACL tears was, by design, impossible. Since the Pac-12 Sports Injury Research Archive database does not include individual-athlete exposure data, we were unable to refine exposure beyond *the athlete-year level*. It must be reemphasized that our definition of *athlete-year*—one athlete appearing on the team roster for a given year—does not necessarily equal a full year of participation in all practices and competitions, since an athlete may become injured, quit the sport, etc., and therefore not complete the entire season. Furthermore, given the variety of playing surfaces available to teams within the same institution, it is not possible to determine true athlete-exposure values across them. Therefore, the IR per athlete-year regarding playing surface reported here (see Table 2) must be interpreted carefully. For example, a higher IR of ACL tears on synthetic versus organic surfaces may be due to greater athlete exposure to synthetic surfaces over the course of 1 year.

## Conclusion

Among our cohort of elite collegiate athletes, most ACL injuries occurred in-season via a noncontact mechanism, and the overall mean IR of ACL injuries per athlete-year was 1.1%. The sport with the highest IR of ACL injury among female athletes was soccer, while among male athletes it was football. Female athletes were observed to have significantly higher IRR of ACL injury than their male counterparts in the sports of softball, gymnastics, and soccer.
